# High-resolution flexible X-ray luminescence imaging enabled by eco-friendly CuI scintillators

**DOI:** 10.3389/fchem.2022.1052574

**Published:** 2022-10-31

**Authors:** Zhongzhu Hong, Peifu Luo, Tingting Wu, Qinxia Wu, Xiaoling Chen, Zhijian Yang, Shuheng Dai, Hao Jiang, Qihao Chen, Qiang Sun, Lili Xie

**Affiliations:** ^1^ MOE Key Laboratory for Analytical Science of Food Safety and Biology, College of Chemistry, Fuzhou University, Fuzhou, China; ^2^ Center for Functional Materials, National University of Singapore Suzhou Research Institute, Suzhou, China; ^3^ School of Public Health, Fujian Medical University, Fuzhou, China

**Keywords:** CuI, microscintillator, radioluminescence, self-trapped exciton, X-ray imaging

## Abstract

Solution-processed scintillators hold great promise in fabrication of low-cost X-ray detectors. However, state of the art of these scintillators is still challenging in their environmental toxicity and instability. In this study, we develop a class of tetradecagonal CuI microcrystals as highly stable, eco-friendly, and low-cost scintillators that exhibit intense radioluminescence under X-ray irradiation. The red broadband emission is attributed to the recombination of self-trapped excitons in CuI microcrystals. We demonstrate the incorporation of such CuI microscintillator into a flexible polymer to fabricate an X-ray detector for high-resolution imaging with a spatial resolution up to 20 line pairs per millimeter (lp mm^−1^), which enables sharp image effects by attaching the flexible imaging detectors onto curved object surfaces.

## Introduction

Digital X-ray imaging has been widely used in medical diagnosis, industrial inspection, and security testing (Kim et al., 2017; Wei and Huang, 2019; Zhao et al., 2020; Wang et al., 2021; Zhou et al., 2022). The past decades have witnessed the rapid development of various scintillators which are capable of converting high-energy X-rays photons to visible signals for indirect X-ray detection (Xu et al., 2020; Ou et al., 2021; Guan et al., 2022; Ma et al., 2022). In particular, inorganic scintillators such as Gd_2_O_2_S:Tb and CsI:Tl were typically used for high-performance radiation detection and X-ray imaging owing to their high X-ray attenuation coefficient, large X-ray conversion efficiency, and large-area fabrication (Mengesha et al., 1998; Nagarkar et al., 1998; Van Eijk, 2002; Weber, 2002). Recently, solution-processable scintillators such as metal-halide perovskites (Chen et al., 2018; Heo et al., 2018; Zhang et al., 2019; Cho et al., 2020; Zhang et al., 2021) and rare-earth-activated fluoride materials (Qiu et al., 2020; Ou et al., 2021; Pei et al., 2021; Chen et al., 2022; Wu et al., 2022) have developed as promising scintillators owing to their tunable radioluminescence and ease of large-area thin-film fabrication. However, many inorganic scintillators still suffer from the issues of rigorous high-temperature fabrication, poor environmental stability, high-cost, and the risk of toxic elements (Li et al., 2016; Lv et al., 2019; Wei et al., 2019; Zhang et al., 2019; Ma et al., 2021). Therefore, it is highly desired to develop solution-processed, highly stable, and eco-friendly scintillators for X-ray imaging.

Cuprous iodide (CuI) has been widely used in organic catalysts, photodetectors, and anode covers (Yang et al., 2017; Byranvand et al., 2018; Yuan et al., 2019; Liu et al., 2021). This high-Z CuI material can be easily synthesized by a solution-processed method and highly stable against environmental moisture, and exhibits strong X-ray absorption and effective broadband self-trapped exciton (STE) emission. Here, we report a facile method for solvothermal synthesis of highly stable tetradecagonal CuI microscintillators with intense X-ray luminescence originating from self-trapped exciton (STE) emission. We further demonstrate the utility of these CuI scintillators to fabricate a flexible detector for high-resolution X-ray luminescence imaging, with a spatial resolution of 20 lp mm^−1^.

## Results and discussion

**FIGURE 1:**
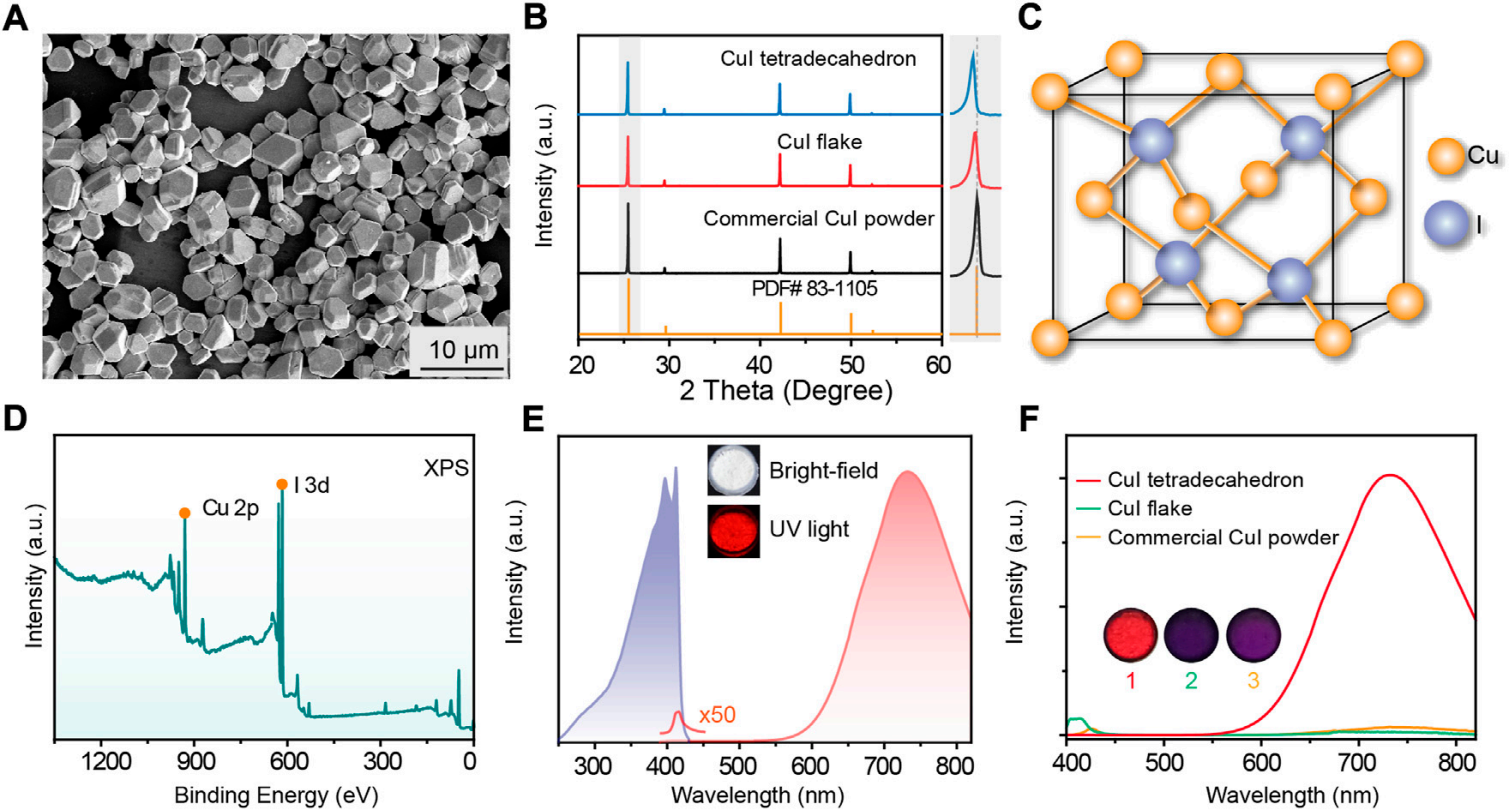
(**A)** SEM image of the as-prepared CuI microcrystals. **(B)** XRD spectra of the as-prepared CuI microcrystals, CuI nanoflakes, commercial CuI powder, and standard CuI PDF card. **(C)** Simplified crystal structural model of the as-prepared CuI microcrystals. **(D)** XPS of the as-prepared CuI microcrystals. **(E)** Excitation spectrum (purple) and emission spectrum (red) of the as-prepared CuI microcrystals. Inset images are photograph of CuI microcrystals power under bright-field and UV excitation, respectively. **(F)** Comparison of fluorescence emission spectra of different CuI samples under UV excitation. Sample #1-3 represent the as-prepared CuI tetradecahedron, CuI flake, and commercial CuI powder, respectively. A same weight of sample powders was used to compare the radioluminescence intensities under the same UV irradiations.

In our experiments, we firstly synthesized sheet-like CuI using a low-temperature coprecipitation method (Shevchenko et al., 2012), which were post-treated by a hydrothermal reaction to obtain better crystallinity of tetradecagonal CuI microcrystals. Scanning electron microscope (SEM) images indicated well-defined tetradecagonal morphologies of the as-synthesized CuI microcrystals with uniform morphology and particle sizes (Figure 1A; Supplementary Figure S1). Powder X-ray diffraction (XRD) measurements showed that the diffraction peaks were well in agreement with the standard CuI (JCPDS#83-1105) (Figure 1B), indicating that the as-synthesized CuI microcrystals have a pure γ-phase sphalerite structure and belongs to the F-43m (216) space group (Figure 1C; Supplementary Table S1) (Chahid and McGreevy, 1998). Scanning X-ray photoelectron spectroscopy (XPS) tests were conducted to confirm the elemental composition and the monovalent iodine and copper in the CuI microcrystals (Figure 1D; Supplementary Figure S2). We further used energy dispersive X-ray spectroscopy (EDS) to confirm uniform distribution of iodine and copper elements in the as-prepared CuI microcrystals (Supplementary Figure S3). The optical spectra of the CuI microcrystals indicated a weak emission at 425 nm and an intense emission at 730 nm under the ultraviolet illumination (Figures 1E,F; Supplementary Figure S4). In addition, a large Stokes shift is also displayed in the as-prepared CuI microcrystals, indicating that these materials are ideal phosphors with almost negligible re-absorption (Figure 1E). The fluorescence emission lifetimes of CuI microcrystals at the emission peak of 425 nm and 730 nm were 1.18 ns and 14.38 μs, respectively (Supplementary Figure S5).

**FIGURE 2:**
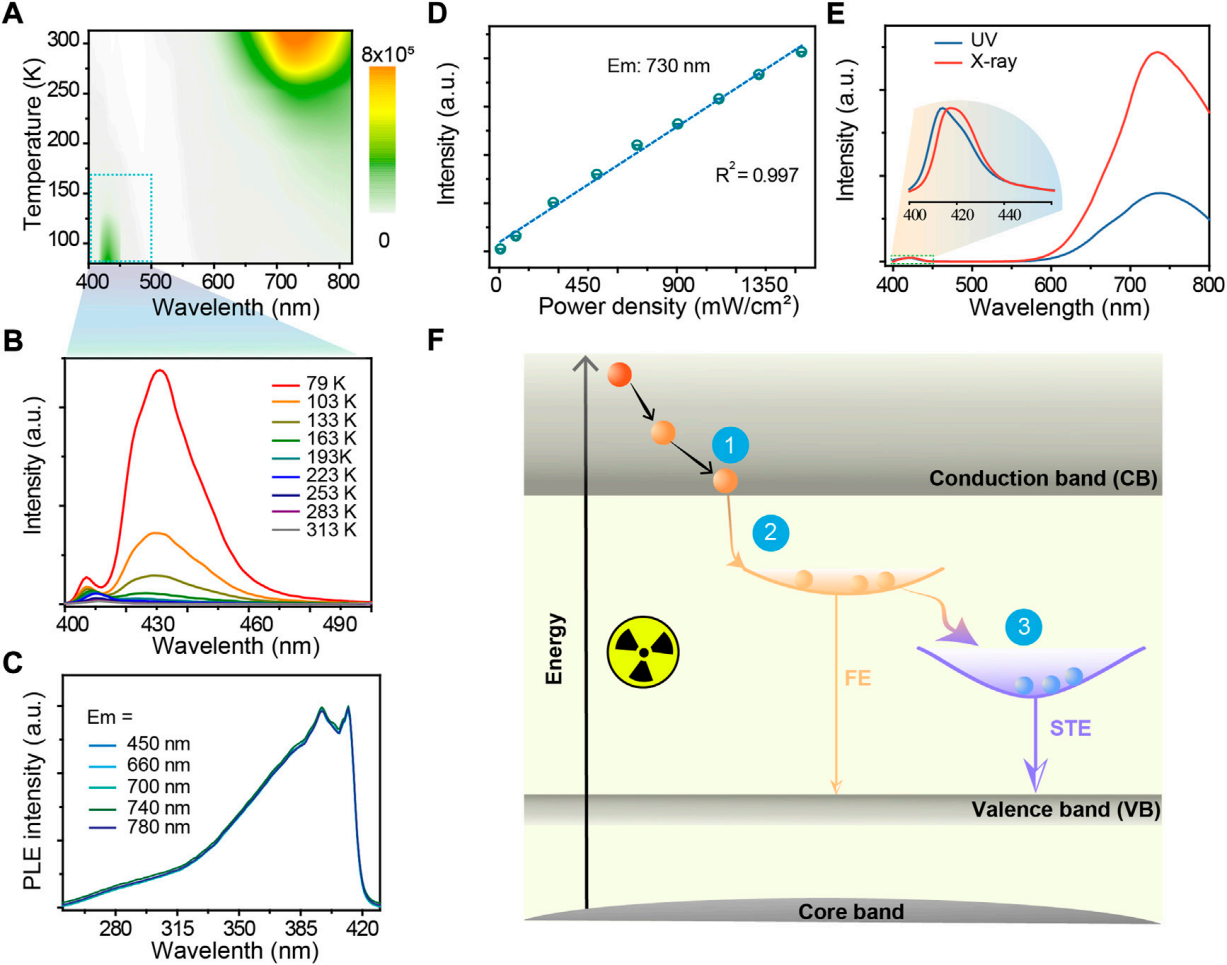
**(A)** Pseudo-color map of temperature-dependent photoluminescence spectra of the CuI microcrystals under UV illumination. **(B)** Temperature-dependent luminescence spectra of CuI microcrystals in the wavelength range of 400–500 nm under UV illumination. **(C)** Excitation spectra at various emissions from the CuI microcrystals. **(D)** Linear relationship between the fluorescence emission intensity and the excitation energy; the excitation wavelength was set at 360 nm **(E)** Fluorescence emission spectra of CuI microcrystals under UV and X-ray excitations, respectively. The data were normalized with the free exciton emission peak. **(F)** Proposed mechanism of X-ray luminescence in CuI microcrystals. Process one is the energy relaxation process, Process two is the process of forming free excitons, and Process three is the exciton self-trapping process.

We performed optical characterization to investigate the photophysical properties of the as-synthesized CuI microcrystals. Temperature-dependent fluorescence spectra showed that the fluorescence emissions at 730 nm decrease with the decreasing of temperature perhaps due to the decrease in exciton-phonon coupling (Figure 2A), while the emission at 425 nm was increased (Figure 2B). EPR spectra showed that the signal at g = 2.003 was increased about 3 times at 298 K and only a little change at 77 K upon UV excitations (Supplementary Figure S6). In addition, the measurement of excitation spectra at various emissions from the CuI microcrystals indicated the same excited states near 400 nm in the CuI microcrystals (Figure 2C; Supplementary Figure S7A). The bandgap in the as-synthesized CuI measured by the UV solid diffuse reflection absorption spectrum was 2.96 eV (Supplementary Figure S7). We further confirmed that fluorescence emission intensity of CuI microcrystals was linearly related with the excitation power (Figure 2D). For these reason, we considered the luminescence emission at 425 nm and 730 nm of the as-prepared CuI microcrystals is possibly dominated by the recombination of free exciton (FE) and self-trapped excitons. Notably, the ratio between the integrated area of the STE emission to the integrated area of the FE emission (S_STE_/S_FE_) increased with the blue-shift of the UV excitation wavelength (Supplementary Figure S8A). In addition, we observed the similar luminescence behavior of CuI microcrystals under X-ray and UV excitation (Figure 2E), suggesting that they possibly originate from the same excited state. Taken together, we reason that the high-Z CuI microcrystal can efficiently absorb the incident X-ray excitations by its host lattice (Figure 2F), and the STE recombinants to produce a broadband emission with a large Stokes shift (Luo et al., 2018; Yang et al., 2019; Lian et al., 2020). This can be further verified by the measured temperature-dependent X-ray luminescence spectra (Supplementary Figure S8B).

**FIGURE 3:**
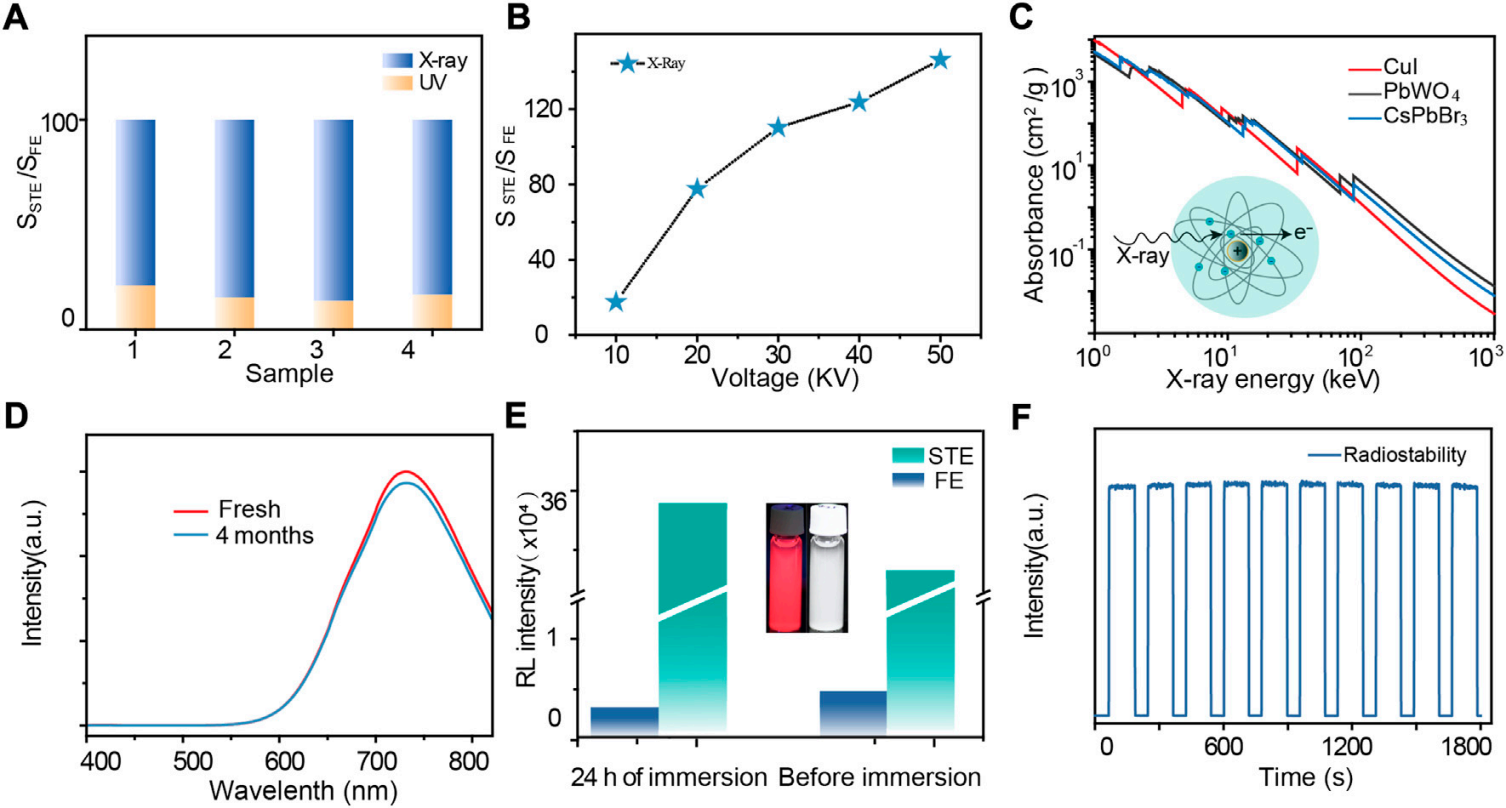
**(A)** Histogram of S_STE_/S_FE_ under UV excitation and S_STE_/S_FE_ under X-ray excitation. **(B)** Absorption spectra of CuI, PbWO_4_ and CsPbBr_3_ as a function of X-ray energy. Attenuation coefficients obtained from reference (Berger et al., 2013). **(C)** The relation between S_STE_/S_FE_ as the function of X-ray tube voltage. **(D)** Comparison of radioluminescence spectra of fresh and 4-month-stored CuI microcrystals. **(E)** STE and FE emission intensities of CuI microcrystals before and after immersion in water. **(F)** Radiation stability of emission at 730 nm under repeated switched X-ray irradiation at a dose rate of 278 μGy s^−1^.

In a further set of experiments, four samples of CuI microcrystals prepared *via* various hydrothermal reaction times (0.5 h, 1 h, 2 h, and 4 h) were used to measure the luminescence intensity of CuI microcrystals under X-ray and UV excitations. It was found that the proportion of STE emission induced by X-ray excitations was much higher as compared to that induced by UV excitations (Figure 3A). By increasing the voltage energy of X-ray excitations, the proportion of STE emission increased correspondingly (Figure 3B). The X-ray absorption coefficient of CuI microcrystals as a function of X-ray photon energy is comparable with other scintillators such as PbWO_4_ and CsPbBr_3_ (Figure 3C), and the radioluminescence intensity of the CuI microcrystals was also comparable with several commercial scintillator powders, including PbWO_4_, Csl:Tl, Bi_4_Ge_3_O_12_, and ZnS:Mn (Supplementary Figure S9). Notably, the merits of low-cost synthesis and nontoxicity of the CuI microcrystals make them more attractive as excellent scintillators for X-ray imaging (Supplementary Figure S10). Moreover, the radioluminescence intensity of these CuI microcrystals only decreased by less than 10% after storage for 4 months (Figure 3D); excellent stability can be maintained even when the material was immersed in water for 24 h (Figure 3E). These CuI microcrystals also exhibited excellent stability against radiation resistance under pulsed X-ray radiation at a dose rate of 278 μGy s^−1^ (Figure 3F; Supplementary Figure S11).

**FIGURE 4:**
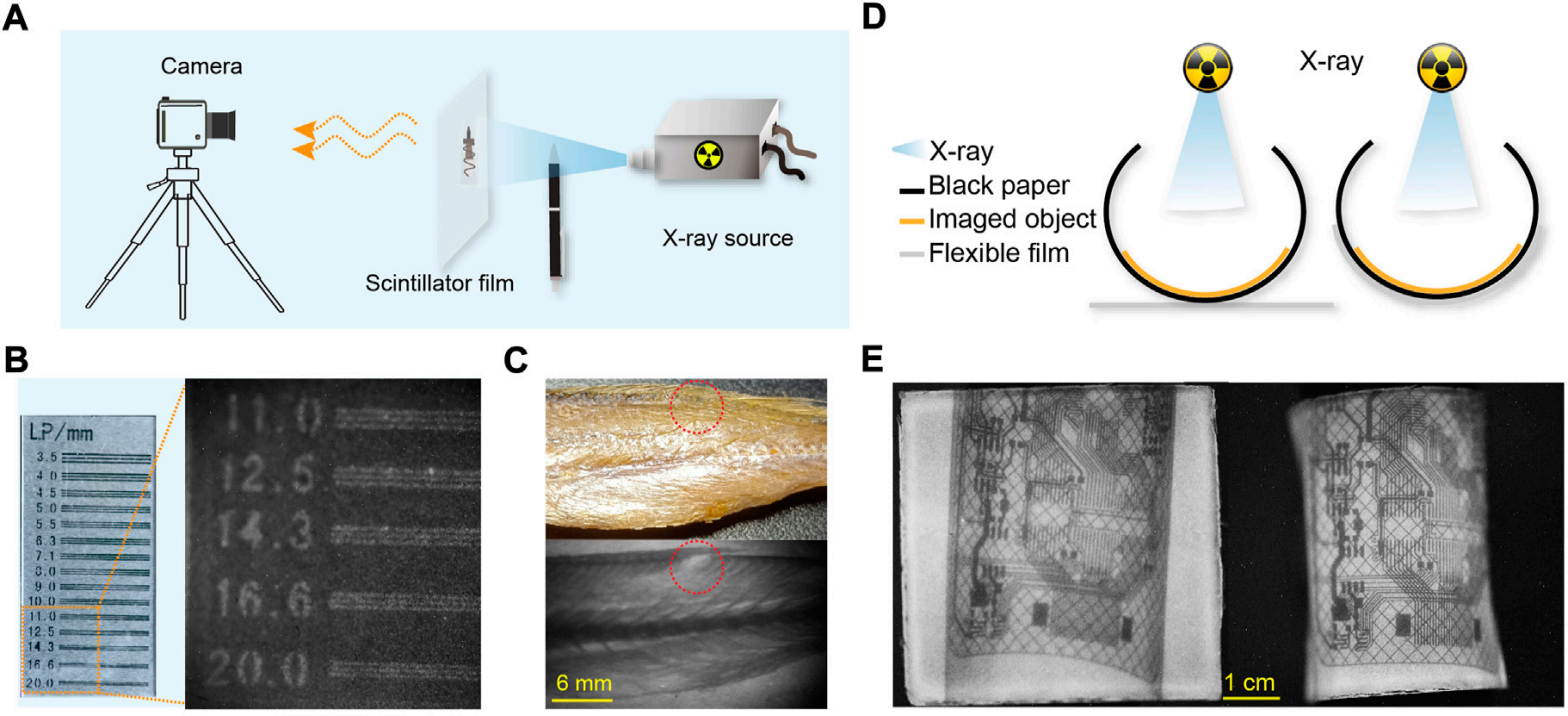
High-resolution, flexible X-ray luminescence imaging. **(A)** Schematic diagram of a home-made imaging system. **(B)** Bright-field image (left) and X-ray imaging (right) of a standard line pair card. **(C)** Bright-field (top) and X-ray imaging (bottom) of a small yellow croaker; the red dotted circle represents the same position. **(D)** Schematic diagram of different imaging modes, including X-ray imaging film attached to the target object or placed outside the target object. **(E)** Plane imaging (left) and curved, flexible imaging (right) of a bend circuit board.

To demonstrate the utility of the CuI microcrystals for X-ray imaging, we further fabricated a flexible and transparent scintillation film by embedding the CuI microcrystals into PDMS elastomers (Supplementary Figures S12, S13). In a typical experiment, the as-fabricated flexible scintillation film was placed between a portable X-ray tube and a digital camera (Figure 4A). The acquired X-ray imaging of a standard line pair card indicated that the spatial imaging resolution was 20 lp/mm (Figure 4B). This flexible detector was further used to perform X-ray imaging of the fine internal structure of a fish, an elastic pen, and others (Figure 4C; Supplementary Figure S14). Moreover, this flexible imaging film can be readily attached onto the curved surface of the imaging object (Figure 4D; Supplementary Figure S15). The experimental results showed that the use of this flexible scintillation film enabled a clear X-ray image with less deformed circuit distribution (Figure 4E; Supplementary Figure S16).

## Conclusion

We have developed a class of high-stability γ-phase CuI microcrystals with uniform tetradecagon morphology and outstanding X-ray luminescence. Our experimental results revealed that the strong broadband radioluminescence of CuI microcrystals originates from efficient X-ray absorption and STE emission. The successful fabrication of CuI microcrystal-embedded flexible X-ray detectors offers a promising technology for high-resolution X-ray imaging of curved objects. Despite the advances in technology, much effort is still required for in-depth understanding X-ray luminescence mechanism and precise control over the materials’ size and morphology of the CuI microcrystals.

## Data Availability

The original contributions presented in the study are included in the article/Supplementary Material, further inquiries can be directed to the corresponding author.
