# Supplementary material for: High-resolution flexible X-ray luminescence imaging enabled by eco-friendly CuI scintillators
Source: Front Chem. 2022 Oct 31;10:1052574. doi: 10.3389/fchem.2022.1052574 (PMC9659724; doi:10.3389/fchem.2022.1052574)
Supplement: Supplementary file 1 [file DataSheet1.PDF]

Supplementary information

High-resolution flexible X-ray luminescence imaging enabled by eco-friendly CuI scintillators

Zhongzhu Hong[a,#], Peifu Luo[a,#], Tingting Wu[a], Qinxia Wu[a], Xiaoling Chen[a], Zhijian Yang[a], Shuheng Dai[a], Hao Jiang[a], Qihao Chen[a], Qiang Sun[b], and Lili Xie[c*]

[a]*MOE Key Laboratory for Analytical Science of Food Safety and Biology, College of Chemistry, Fuzhou University, Fuzhou 350108, P. R. China*
[b]*Center for Functional Materials, National University of Singapore Suzhou Research Institute, Suzhou 215123, China*
[c]*School of Public Health, Fujian Medical University, Fuzhou 350122, P. R. China.*

[*]Corresponding author E-mail: 1006xielili@fjmu.edu.can

1. Experiment section

1.1 Materials

Copper(II) sulfate pentahydrate ($CuSO_4·5H_2O$, 98.0%), and sodium iodide (NaI, 99.5%) were purchased from Sigma-Aldrich. Sodium sulfite (Na_2SO_3, 97%) was purchased from Amethyst chemcas. All chemicals were used without any purifications.

1.2 Synthesis of CuI micron crystals

In a typical synthesis procedure, the same moles of $CuSO_4·5H_2O$, NaI, and Na_2SO_3 were dissolved in deionized water to form clear solutions, respectively. Subsequently, the solutions of $CuSO_4·5H_2O$, NaI, and Na_2SO_3 were sequentially added to the beaker for reaction to obtain a suspension. The suspension obtained from the reaction was added to a high-pressure polytetrafluoroethylene liner, sealed in a stainless steel autoclave, and then placed in an oven at 200 ºC for 4 h. Microcrystals were precipitated by slowly cooling the solution down to room temperature. Finally, the obtained microcrystals were filtered out, washed with water and ethanol, and dried at 60 °C overnight.

2. Measurements and characterizations

X-ray powder diffractometer (DY1602/Empyrean) was used to characterize the X-ray diffraction analysis of the samples in the range of 10-80°. X-ray photoelectron spectroscopy

(XPS) was measured with X-ray photoelectron spectroscopy (ESCALAB 250). Steady-state and time-resolved luminescence spectra were recorded by a spectrometer (FLS 980 Edinburgh Instruments Ltd.). The integrated intensity of the radioluminescence under X-ray radiation, and the radioluminescence stability were recorded by an Edinburgh FS5 fluorescence spectrophotometer equipped with a mini X-ray tube. The morphology analysis and element mapping of the as-synthesized CuI microcrystals were performed by a high-resolution field emission scanning electron microscope (SEM) (Verios G4). The pictures and imaging photos of various samples were recorded by Nikon camera D850. The ultraviolet solid diffuse reflection was tested using a UV-visible spectrophotometer (LAMBDA800 PE). The EPR signal was recorded with a Bruker model A300 spectrometer at a temperature ranging from 77 K to 298 K. The synchronous thermal analyzer (STA449C/6/G) was used to perform thermogravimetric (TG) analysis and differential scanning calorimetry (DSC) analysis in a N_2 environment.

3. Fabrication of flexible scintillation films

A certain amount of CuI powder dispersed in cyclohexane was mixed with 1.5 g of Dow Corning 184 silica gel matrix, and 0.15 g of curing agent was further added for mixing. The mixed precursor was poured into a square Acrylic template, following heated at 80 °C for 5 h, and then cooled to room temperature for obtaining a flexible scintillation film.

Table S1. Single-crystal X-ray diffraction data of CuI single crystals.

CuI	Ref[1]	This work
Space group	F43m	
a (Å)	6.054	6.052
b (Å)	6.054	6.052
c (Å)	6.054	6.052
α (°)	90	90
β (°)	90	90
γ (°)	90	90
cell volume (Å^3)	221.940	221.667

Table S2. XPS analysis of CuI microcrystals.

Name	Peak /eV	FWHM /eV	Area CPS/eV	Atomic/%
Cu 2p	929.91	2.95	849554.66	47.24
I 3d	616.66	1.80	1367203.35	52.76

Table S3. EDS analysis of elements in the CuI microcrystals.

No.	Cu (Atomic %)	I (Atomic %)	Total (Atomic %)
1	48.34	51.66	100
2	49.45	50.55	
3	47.48	52.52	
Average	48.42	51.58	

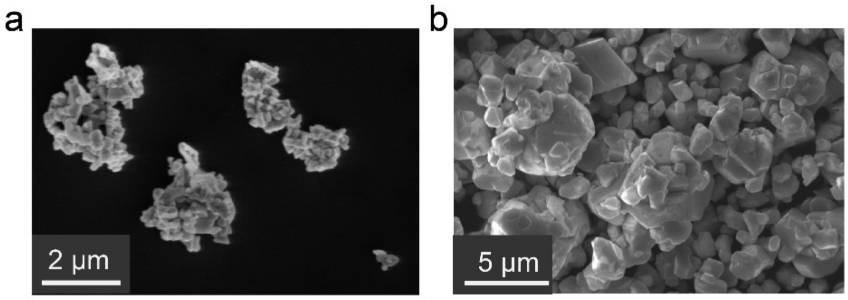

Figure S1. (a) SEM image of CuI nanoflakes. (b) SEM image of commercial CuI power.

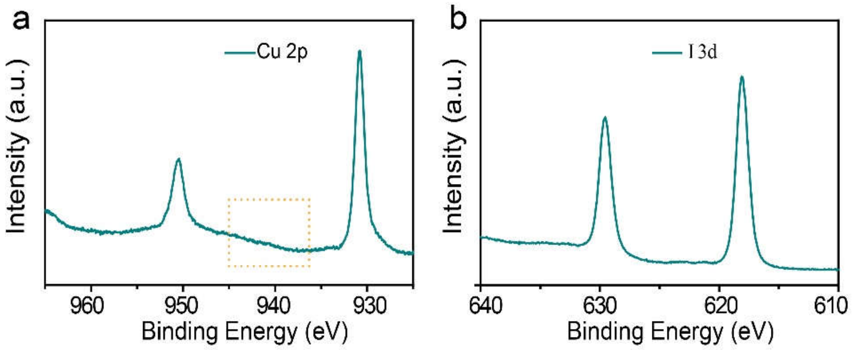

Figure S2. (a) X-ray photoelectron spectroscopic data of Cu 2p.（b）X-ray photoelectron spectroscopic data of I 3d.

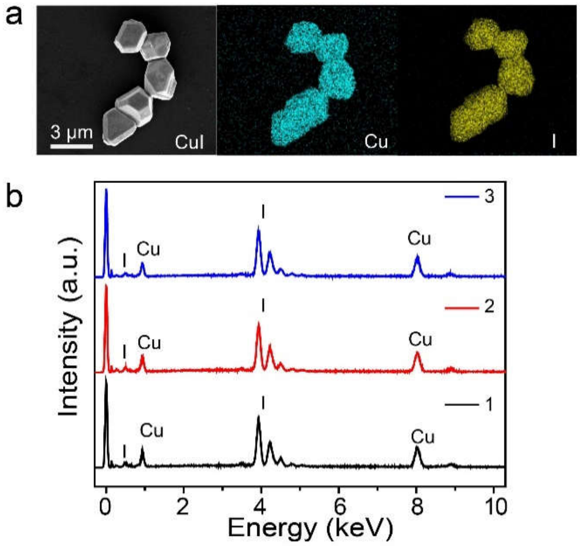

Figure S3. (a) Elemental mapping of CuI microcrystals. (b) Elemental semi-quantitative analysis of CuI in three different regions.

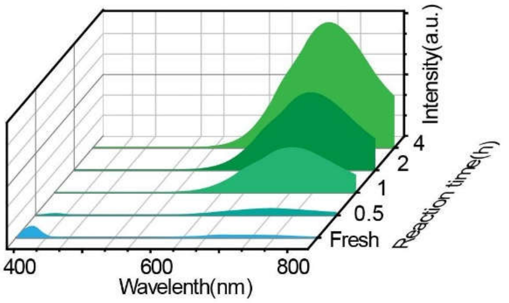

Figure S4. Luminescence spectra of CuI microcrystals treated with different hydrothermal reaction times. The hydrothermal reaction times were 0 h, 0.5 h, 1 h, 2 h, and 4 h, respectively.

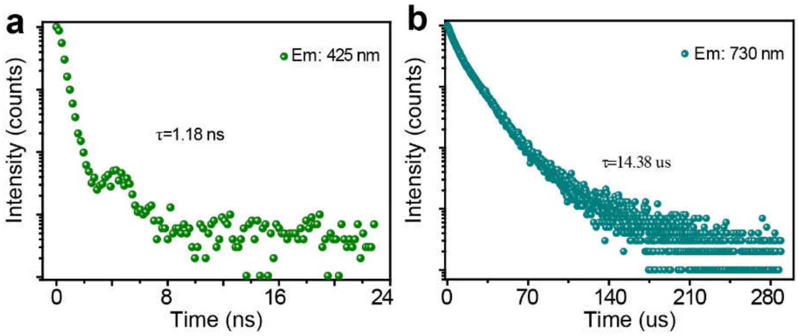

Figure S5. Time-resolved luminescence decay curve at the emission wavelength of (a) 425 nm and (b) 720 nm.

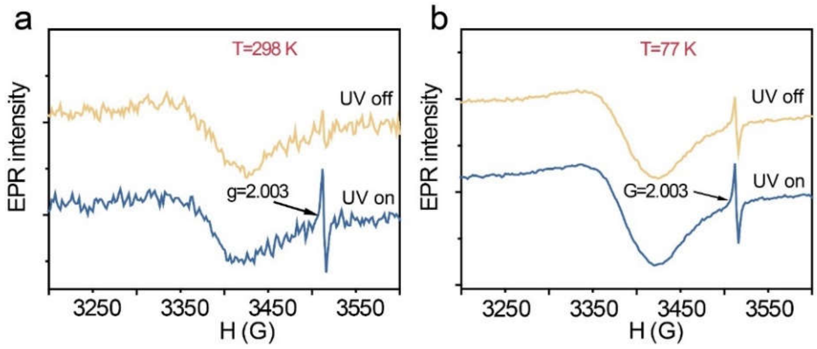

Figure S6. The electron paramagnetic resonance (EPR) of CuI microcrystals. (a) EPR spectra tested at 298 K with UV on or off. (b) EPR spectra tested at 77 K with UV on or off.

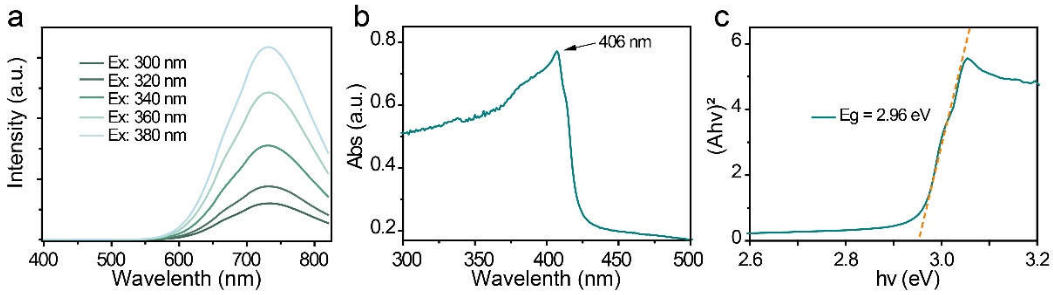

Figure S7. (a) Fluorescence emission spectra of CuI microcrystals excited with different UV wavelengths. (b) Solid UV diffuse reflectance absorption spectra of CuI microcrystals. (c) Optical bandgap width of CuI microcrystals.

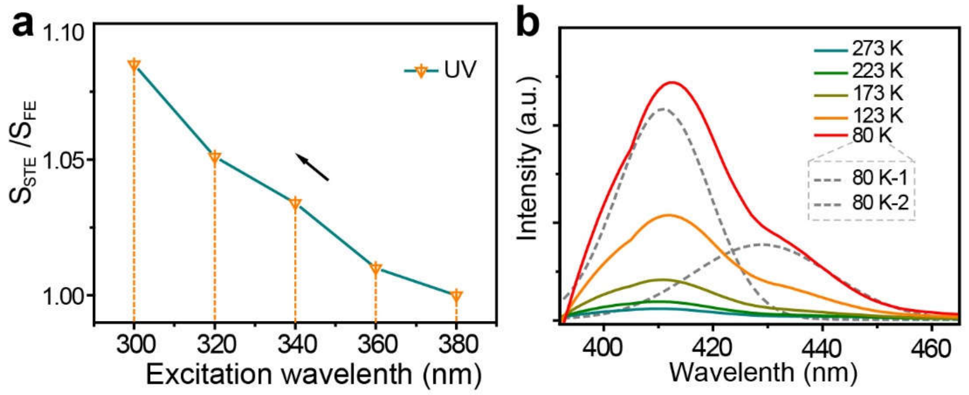

Figure S8. (a) Curve of the ratio of the integrated area of the STE emission peak to the free exciton (FE) emission peak (S_{STE}/S_{FE}) as a function of excitation wavelength. (b) X-ray fluorescence spectra recorded at different temperatures. The dotted was the Gaussian peak fitting of the spectral line at 80 K.

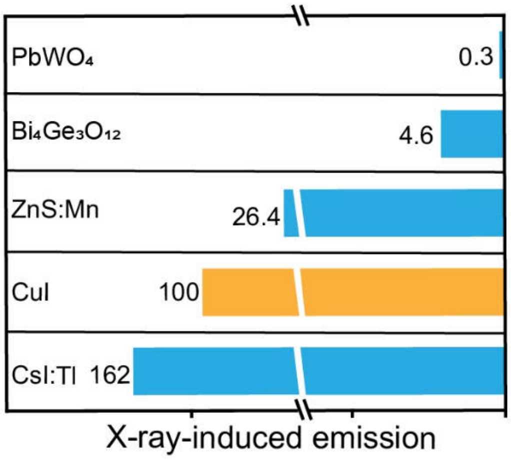

Figure S9. Comparison of the radioluminescence intensity of various scintillator materials under X-rays (50 kV). Same weight of sample powders was used to compare the radioluminescence intensities under the X-ray irradiations.

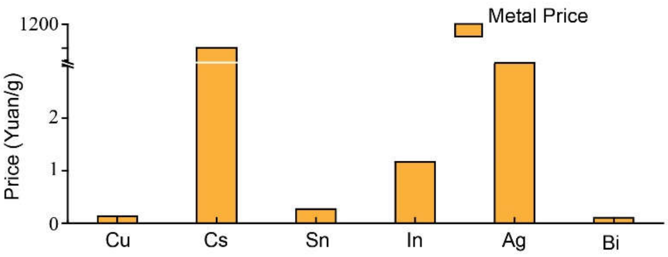

Figure S10. Prices of various metal elements[2]. The data were recorded on July 8, 2022.

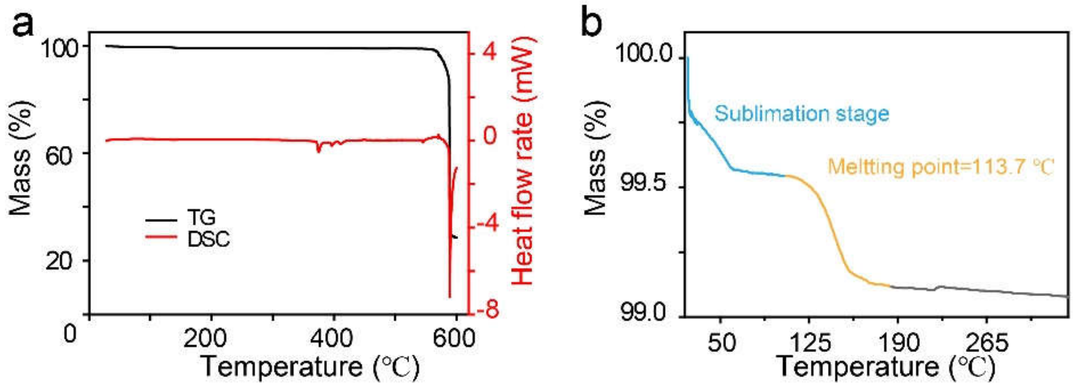

Figure S11. (a) TG and DSC curve of CuI microcrystals. (b) Two plateaus of mass drop between 30-200 °C in the TG plot.

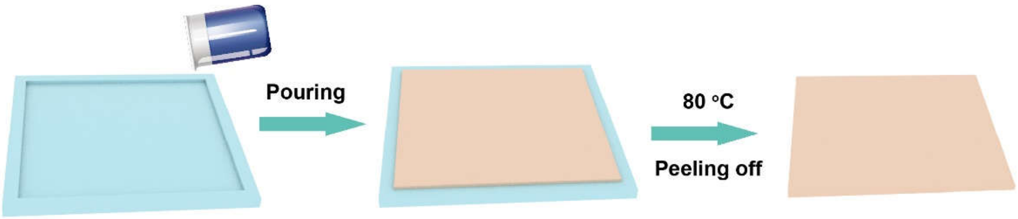

Figure S12. Schematic diagram of the process for the preparation of flexible X-ray imaging film by embedding the CuI microcrystals into PDMS elastomers.

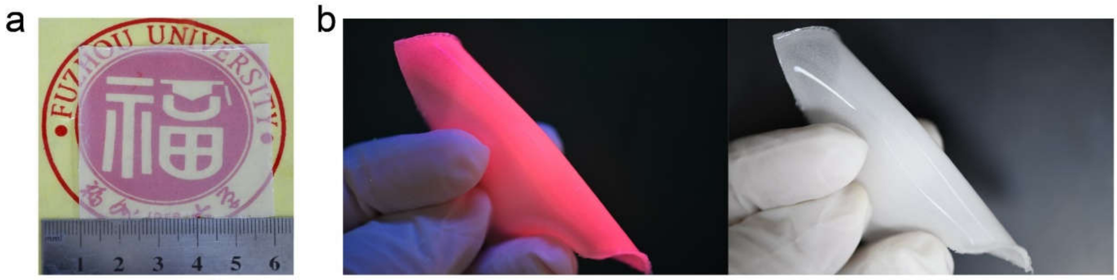

Figure S13. (a) Transparency of the as-prepared CuI-embedded flexible scintillation film. (b) The left and right panels are photographs of the curved film under UV excitation and brightfield, respectively.

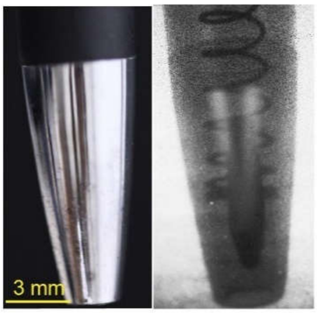

Figure S14. Bright-field photograph and X-ray image of the spring pen; the scale bar is 3 mm.

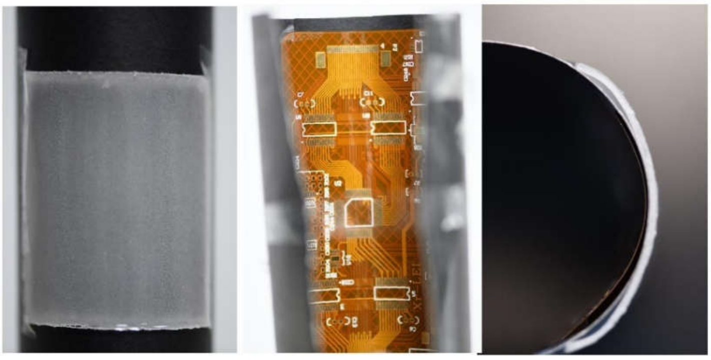

Figure S15. Curved patterns made of flexible circuit boards. The flexible scintillator film can be bent and attached to the curved pattern.

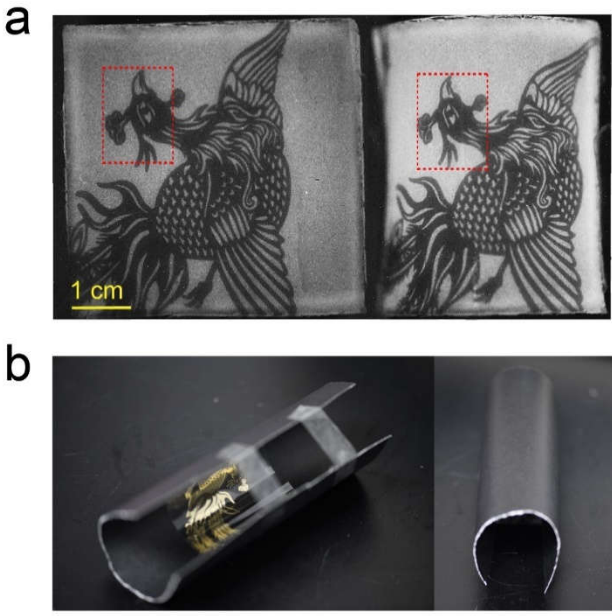

Figure S16. (a) The left image was the plane imaging of the curved phoenix pattern, and the right image was the curved surface imaging of the curved phoenix pattern. The red dotted box was the same size, and the phoenix pattern in the planar imaging was obviously deformed. (b) Bright-field photograph of the curved phoenix pattern.

References

[1] Keen S.H., (1994). High-pressure polymorphism of the copper (I) halides: A neutron-diffraction study to ~10 GPa [J]. *Phys. Rev. B*, 50(9), 5868. doi: 10.1103/physrevb.50.5868.

[2] Changjiang nonferrous (Xiamen) technology Co. L., (2022). Metal futures price, https://www.ccmn.cn/.
